# Application of Systems Biology and Bioinformatics Methods in Biochemistry and Biomedicine

**DOI:** 10.1155/2013/651968

**Published:** 2013-12-31

**Authors:** Yudong Cai, Tao Huang, Lei Chen, Bin Niu

**Affiliations:** ^1^Institute of Systems Biology, Shanghai University, Shanghai 200444, China; ^2^Department of Genetics and Genomics Sciences, Mount Sinai School of Medicine, New York, NY 10029, USA; ^3^College of Information Engineering, Shanghai Maritime University, Shanghai 201306, China; ^4^College of Life Science, Shanghai University, Shanghai 200444, China

With the explosively increasing high-throughput omics data, it is highly desired to develop effective computational methods and tools that can mine useful information to support the development of biochemistry, biomedicine, and drug design. Furthermore, in order to understand the protein-protein, protein-D/RNA, and other complex interactions, systems biology approaches are applied.

In this collection, diverse topics were covered and there are many novel methods and intriguing findings.

Y. Jiang et al. compared the gene expressions among the colorectal cancer patients in different stages and obtained the early and late stage biomarkers. Then, these two kinds of biomarkers were both mapped onto the protein interaction network, and the signal propagation path from the early stage biomarker to the late one was identified. Their findings may provide useful insights for revealing the mechanism of colorectal cancer progression at the cellular systems biology level.

L. N. Lili et al. investigated the process of stroma activation in human ovarian cancer by molecular analysis of matched sets of cancer and surrounding stroma tissues. They found that functionally significant variability exists among ovarian cancer patients in the ability of the microenvironment to modulate cancer development.

B. Yang et al. constructed a network-based inference framework for identifying cancer genes from gene expression data. Six identified genes (TSPYL5, CD55, CCNE2, DCK, BBC3, and MUC1) susceptible to breast cancer were verified through the literature mining, GO analysis, and pathway functional enrichment analysis.

Lung cancer is one of the most malignant cancers. B. Q. Li et al. identified 25 NSCLC and 38 SCLC genes with the shortest path approach in PPI networks. These candidate genes contained more cancer genes and more functional similarity with cancer genes than those identified from the gene expression profiles.

A. R. Iskandar et al. evaluated the perturbation of xenobiotic metabolism in response to cigarette smoke exposure in nasal and bronchial tissues. Their observation suggested that the effects of cigarette smoke exposure on the xenobiotic responses in the bronchial and nasal epithelium of smokers were similar to those observed in their respective organotypic models exposed to cigarette smoke, and nasal tissue could be a used as a reliable surrogate to measure the xenobiotic responses in the bronchial tissue.

E. G. Maiorov et al. identified interconnected markers for T-cell acute lymphoblastic leukemia (T-ALL). Their identified genes may serve as biomarkers, alternative to the traditional ones used for the diagnosis of T-ALL, and help understand the pathogenesis of the disease.

M. Kalita et al. used a multiplex gene expression profiling platform to investigate the perturbations of the innate pathways induced by TGF in a primary airway epithelial cell model of epithelial mesenchymal transition (EMT). Their results indicated that epigenetic changes produced by EMT induce dynamic state changes of the innate signaling pathway.

C. Lu et al. studied the functions of microRNAs related to the liver regeneration of the whitespotted bamboo shark, *Chiloscyllium plagiosum*. Their work deepened the understanding of mechanisms of liver regeneration and resulted in the addition of a significant number of novel miRNAs sequences to GenBank.

T. Alioto et al. presented a lightweight pipeline for first-pass gene prediction on newly sequenced genomes. The two main components are ASPic, a program that derives highly accurate, albeit not necessarily complete, EST-based transcript annotations from EST alignments. The other component is GeneID, a standard gene prediction program, which we have modified to take as evidence intron annotations. The pipeline was successfully tested on the entire C. elegans genome and the 44 ENCODE human pilot regions.

J. Zou et al. reviewed advanced systems biology methods in drug discovery and translational biomedicine. Their review provided a framework for addressing disease mechanism and approaching drug discovery.

L. Chen et al. proposed a computational method to predict the side effects of drugs, which integrated the information of chemical-chemical and protein-chemical interactions. Compared to most of the previous studies, the proposed method can provide the order information of the side effects for any query drug.

K. Wang et al. proposed an accurate method for protein-ligand binding site on protein surface using SVM and statistical depth function. The accuracy, sensitivity, and specificity on training set are 77.55%, 56.15%, and 87.96%, respectively, and on the independent test set the accuracy, sensitivity, and specificity are 80.36%, 53.53%, and 92.38%, respectively.

K. K. Tseng et al. presented a new system and novel approaches to classify different kinds of sperm images in order to assess their health. In their evaluation, the method reached accuracy of 87.5% and has better performance than the existing approaches to sperm classification.

A rapid method is required to mitigate complexity and computation challenges on high throughput protein identification. In Method for Rapid Protein Identification in a Large Database, an accelerated open method is presented by W. Zhang et al. to satisfy this requirement to some extent.

Q. Zou et al. proposed a novel method for distinguishing cytokine from other proteins. It is of vital importance of identifying cytokine in silicon. Ensemble classification strategy was employed for improving the prediction performance, and a friendly prediction web server was also developed.

Du and Yu introduced a novel method, SubMito-PSPCP, which embeds the PSSM into the pseudoamino acid compositions, to predict protein submitochondrial locations.

T. Gu et al. applied the Support Vector Regression and a two stage feature selection to developing the computational model which maps DPP-IV inhibitors to the activity. They also developed the online server.

Based on nonlinear mapping and Coulomb function, X. Liu et al. applied 3D kernel approach to predict the four protein tertiary structural classes and five membrane protein types with satisfactory results. It has not escaped our notice that kernel approaches may hold a high potential for predicting the other protein features.

T. H. Zhao et al. proposed a new method to predict protein disordered regions based on sequence features. The accuracy and MCC (Matthew's correlation coefficient) of their method are higher than three popular disordered region predictors: DISOPRED, DISOclust, and OnD-CRF.

M. S. M. Ali et al. studied the structure and function of LipA8 which is able to adapt to extreme temperatures. Simulations show that it is most stable at 0°C and 5°C. In extreme temperature, the catalytic domain (N-terminus) maintained its stability than the noncatalytic domain (C-terminus), but the noncatalytic domain showed higher flexibility than the catalytic domain.

A Boolean network (BN) is widely used as a model of gene regulatory networks. K. Kobayashi et al. proposed a BN model with two types of the control inputs and an optimal control method with duration of drug effectiveness. The optimal control problem is reduced to an integer programming problem.

J. Zhang et al. studied the microRNA-mediated regulation in biological systems with oscillatory behavior. They started with two specific microRNA-mediated regulatory circuits which show their fine-tuning roles in the modulation of periodic behavior and then applied these results to study the effects of miR369-3 regulation of cell cycle.

B. Yan et al. developed a mathematical model to study the mechanisms underlying the size checkpoint in fission yeast. They found that when the spatiotemporal regulation is coupled to the positive feedback loops, the mitosis-promoting factor (MPF) exhibits a bistable steady-state relationship with the cell size. The switch-like response from the positive feedback loops naturally generates the cell size checkpoint.

Detection of potential siRNA off-targets is crucial for High Content Screening (HCS) using small interfering RNAs (siRNAs). S. Das et al. performed a detailed off-target analysis of three most commonly used kinome siRNA libraries based on latest RefSeq version and created SeedSeq database, a new unique format to store off-target information.

L. Zhu et al. systematically investigated the characteristics and evolutionary pattern of actin gene family in primates. Phylogenetic analysis of 233 actin genes in human, chimpanzee, gorilla, orangutan, gibbon, rhesus monkey, and marmoset genomes showed that actin genes in the seven species could be divided into two major types of clades: orthologous group versus complex group. Codon usages and gene expression patterns of actin gene copies were highly consistent among the groups because of basic functions needed by the organisms but much diverged within species due to functional diversification.

J. Ping et al. performed long time-scale molecular dynamics simulations on both open and closed states of *Escherichia coli* adenylate kinase (ADK); based on which a conformational selection mechanism was proposed to explain the large scale domain motion of this enzyme.

*Yudong Cai* *Tao Huang* *Lei Chen* *Bin Niu*

